# *Clostridium butyricum* Helps to Alleviate Inflammation in Weaned Piglets Challenged With Enterotoxigenic *Escherichia coli* K88

**DOI:** 10.3389/fvets.2021.683863

**Published:** 2021-07-02

**Authors:** Haihua Li, Xuejiao Liu, Zhiyuan Shang, Jiayun Qiao

**Affiliations:** ^1^Tianjin Key Laboratory of Agricultural Animal Breeding and Healthy Husbandry, College of Animal Science and Veterinary Medicine, Tianjin Agricultural University, Tianjin, China; ^2^Tianjin Key Laboratory of Animal and Plant Resistance, College of Life Sciences, Tianjin Normal University, Tianjin, China

**Keywords:** inflammatory response, *Clostridium butyricum*, enterotoxigenic *Escherichia coli* K88, weaned piglet, TLRs/NF-κB

## Abstract

**Background:** Whether the probiotic *Clostridium butyricum* (CB) alleviates enterotoxigenic *Escherichia coli* (ETEC) K88-induced inflammation by regulating the activation of the toll-like receptor (TLR) signaling pathway is not clear, thus, we carried out this study. A total of 72 piglets (average body weight 7.09 ± 0.2 kg) were randomly divided into three groups of 24 piglets per group. Pigs were either fed a daily diet (NC, negative control), a diet tested every day by 1 × 10^9^ CFU/mL ETEC K88 (PC, positive control), or a basal diet supplemented with 5 × 10^5^ CFU/g CB and challenged with ETEC K88 (PC + CB group).

**Results:** Our results showed that CB pretreatment attenuated the effect of ETEC K88 by decreasing C-reactive protein (CRP), which resulted in tumor necrosis factor alpha (TNF-α) and interleukin-6 (IL-6) production. Histological examination revealed that CB pretreatment alleviated intestinal villi injury caused by ETEC K88 challenge. Furthermore, CB pretreatment promoted mRNA expression of the negative regulators of TLR signaling, including myeloid differentiation factor (MyD88), toll-interacting protein (Tollip), and B cell CLL/lymphoma 3 (Bcl-3), in the intestines of ETEC K88-challenged piglets. ETEC K88-induced activation of nuclear factor kappa B (NF-κB) and nuclear factor of kappa light polypeptide gene enhancer in B cells inhibitor alpha (IκBα) was attenuated by CB pretreatment.

**Conclusion:** These findings indicate that CB helps to maintain and strengthen the shape of intestinal villi and limits detrimental inflammatory responses, partly by inhibiting toll-like receptor 2 (TLR-2), toll-like receptor 4 (TLR-4), and toll-like receptor 5 (TLR-5) expression and inhibiting NF-κB p65, and promoting IκBα activation and synergism among its negative regulators.

## Introduction

Inflammation is a fundamental aspect of the health and function of the intestinal tract. A healthy intestinal tract should be in a constant state of “controlled” inflammation (1). However, when compared with a germ-free pig, the intestine of a conventional pig displays markedly upregulated expression of pro-inflammatory cytokines (2). When overt intestinal infections, such as ETEC or *Salmonella typhimurium* infections, occur, inflammatory responses are drastically amplified and intestinal morphology and function are further impaired (3–5). In addition, inflammation induced by stresses, such as weaning, also has a substantial impact to the intestine (6, 7). Although moderate inflammation is part of the physiological mechanism of combating infection, unbridled inflammatory response will cause tissue damage (8). *Clostridium butyricum* (CB) is a butyric acid-producing spore-forming, gram-positive anaerobe bacterium that is found in the soil and intestines of healthy animals and humans (9, 10). CB can survive in conditions of low pH and relatively high bile concentrations and produce endospores. CB has been shown to increase the resistance of the gut to pathogen invasion via inducing the secretion of anti-inflammatory cytokines in the intestine (1). These properties of CB lend it to being used as a probiotic and make it suitable as a probiotic supplement in animal feed (11). CB has, therefore, become increasingly important in preventing and treating intestinal inflammation or inflammation-related diseases (12).

CB carries bacterial antigens that can stimulate the body to produce a moderate immune response, which in turn can protect the gut against pathogen invasion (13). Additionally, CB promotes the proliferation of cecal *Lactobacillus*, which increases villus height and decreases crypt depth in the jejunum of enterotoxigenic *Escherichia coli* (ETEC)-challenged broiler chickens (14, 15). *In vivo*, moderate levels of pro-inflammatory cytokine production were observed in silvery pomfret intestinal epithelial cells treated with CB (16). *In vitro*, the pretreatment of HT-29 cells with CB attenuated the expression of cytokines following inflammation induced with *S. aureus* lipoteichoic acid (17). Furthermore, CB prevents acute experimental colitis in mice by inducing the production of interleukin-10 (IL-10), which is an anti-inflammatory cytokine (18). Finally, CB maintains the balance between pro-inflammatory and anti-inflammatory cytokine production in patients with inflammatory bowel disease (IBD) (19). However, the exact mechanism underlying the probiotic modulation of the inflammatory response remains unclear.

Pattern recognition receptors, such as toll-like receptors (TLRs), are important in the detection of microbial infection and the induction of inflammatory and immune responses by recognizing pathogen-associated molecular patterns (PAMPs) (14). Myeloid differentiation factor, MyD88, is a major cytoplasmic adaptor protein that is shared by the TLR family, except for TLR-3, and has been implicated in signaling responses to a variety of PAMPs (20). Functional active nuclear factor kappa B (NF-κB) exists as a homodimer or heterodimer in cells that consists of p65 and p50 subunits, and is normally inactive in the cytoplasm by binding to the NF-κB inhibitory protein (IκB) (21). The activation of TLRs triggers the NF-κB and nuclear factor of kappa light polypeptide gene enhancer in B cell inhibitor alpha (IκBα) marking pathways and prompts, which stimulate the combining of cytokines and chemokines such as tumor necrosis factor alpha (TNF-α), interleukin-6 (IL-6), interleukin-1β (IL-1β), and interleukin-8 (IL-8) (22–25). Consequently, inflammatory mediators mediate the host defense against invading pathogens as well as cause elicit collateral host-tissue injury (26). TLR signaling plays a regulatory role by maintaining a balance between the activation and inhibition of the immune system to prevent detrimental and inappropriate inflammatory responses (24, 25, 27). Various inhibitory factors of TLR signaling, including toll-interacting protein (Tollip), interleukin−1 receptor combined with kinase M (IRAK-M), B cell CLL/lymphoma 3 (Bcl-3), and peroxisome proliferator-activated receptor-γ (PPARγ), have been identified and characterized, particularly in terms of chronic inflammatory together with potentially detrimental TLR resorted to PAMPs (28).

To our knowledge, the molecular mechanism of CB regulating the intestinal health of piglets through the inflammatory pathway is still not clear. Thus, we hypothesized that the administration of the probiotic CB alleviates ETEC K88-induced inflammation by regulating the activation of the TLR signaling pathway. The aim of the present study was to investigate the ability of CB to alleviate ETEC K88-induced inflammation and to characterize its effects on the associated morphological disruption of the intestine and the activation of TLR signaling in the intestines in response to infection. This study provides molecular evidence that further supports the findings of previous reports showing that the application of CB assists in alleviating inflammation following intestinal infections in piglets.

## Materials and Methods

All the procedures mentioned in this research were provided by the Institutional Animal Care and Use Committee of Tianjin Normal University (Tianjin, China) (No: TNU012).

### Bacterial Strains

The China General Microbiological Culture Collection Center (CGMCC13951) provided data for CB. Moreover, the China Veterinary Culture Collection Center (CVCC1502) provided ETEC K88. CB and ETEC K88's collection and calculation have been mentioned before (29).

### Animals, Experimental Design, and Diets

Incubation for 114 days was the prerequisite for the research subjects. The experimental period lasted 21 ± 2 days. A total of 72 crossbred (Duroc × Landrace × Yorkshire) healthy female piglets were fed a corn-soybean meal-fish meal daily diet that was formulated to approximately meet the National Research Council (NRC 2012) (Table 1) requirements for all nutrients. Prior to the start of the trial, no clinical signs of diarrhea or other diseases were observed in any of the piglets.

**Table 1:** Ingredients and chemical composition of the basal diet (%w/w, as-fed basis)^a^.

**Item**	**Amount**
**Ingredient, %**	
Corn, yellow	63.20
Soybean meal, 43% CP (crude protein)	19.00
Whey powder	4.80
Fish meal, 65% CP	8.60
Glucose	1.00
Acidifier	0.30
Calcium hydrogen phosphate	0.60
Limestone	0.70
Salt	0.30
L-Lys • HCL, 78% Lys	0.30
DL-Met, 99% Met	0.10
L-Thr, 98% Thr	0.10
Vitamin and mineral premix^a^	1.00
**Calculated composition**	
DE (digestible energy), Mcal/kg	3.25
Lys, %	1.39
Met, %	0.53
**Analyzed composition**	
Crude protein	18.75
Crude fat	3.42
Calcium	0.88
Total phosphorus	0.71
Crude fiber	2.20

a *Supplying a minimum per kilogram complete diet of: 12,500 IU Vitamin A; 1,250 IU Vitamin D; 125 IU Vitamin E; 90 μg Vitamin B12; 10 mg riboflavin; 48 mg pantothenic acid; 35 mg niacin; 4.5 mg folic acid; 0.25 mg biotin; 130 mg Fe; 180 mg Zn; 15 mg Cu; 30 mg Mn; 0.60 mg I, and 0.25 mg Se*.

The weight of the 72 piglets was 7.09 ± 0.2 kg on average. A total of 24 piglets were divided into each group randomly. Each group was given the following different treatments: (1) piglets were fed with sterile normal saline (NC, control group); (2) piglets were fed with ETEC K88 (PC, ETEC group); and (3) piglets were fed with CB and ETEC K88 (PC + CB). The feeding and management of piglets refer to the methods previously reported (29). NC and PC received the daily diet at 28 days of age throughout the 14 days of the feeding trial, while the PC + CB group received the daily diet supplemented with 5 × 10^5^ CFU/g CB. ETEC K88 (1 × 10^9^ CFU/mL) was adulterated with sterile normal saline. On day 15, PC and PC + CB were orally administered with ETEC K88 at a dose of 1 × 10^9^ CFU/kg body weight. The NC group received sterile physiological saline (1 mL/kg BW). The dose of ETEC K88 was chosen as noted previously and could cause obvious pathological changes at 24 h post challenge (29).

### Samples Collection and Processing

Six healthy piglets were selected randomly from each treatment group for blood sample collection from the frontal vena cava using coagulation-promoting vacuum tubes (Becton Dickinson Vacutainer Systems, FranklinLakes, NJ, USA) at 3, 6, and 12 h post challenge. Sera were obtained after centrifugation (3,000 × g, 15 min) at room temperature, and then stored at −80°C in Eppendorf tubes until the analysis determining the concentrations of CRP, TNF-α, and IL-6.

At 3, 6, and 12 h post challenge, the piglets whose blood was collected were euthanized and the middle jejunum segments were immediately collected and gently washed with sterile physiological saline. One part was immediately frozen in liquid nitrogen and stored at −80°C for subsequent mRNA and protein analysis, whereas the other was immediately fixed in 4% neutral formalin solution for histopathological examination.

### ELISA

The serum CRP was determined by the commercial pig specific ELISA Kit (Jiancheng Bioengineering Institute, Nanjing, China), and another ELISA Kit (R and D system, Minneapolis, MN) was used for serum TNF-α and IL-6. The detection limits of CRP, TNF-α, and IL-6 were 0.5 μg/mL, 5 pg/mL, and 4.3 pg/mL, respectively.

### Real-Time Quantitative PCR

Based on the manufacturer's instructions, Trizol (Invitrogen, Carlsbad, CA, USA) was used to extract total RNA from tissue samples. The ratio of OD260: OD280 was 1.8-2.0 in all samples. The integrity of RNA was detected by agarose gel electrophoresis. Based on the manufacturer's guidelines, Moloney murine leukemia virus (Promega, Madison, WI, USA) was used to reverse transcribe RNA samples into complementary DNA.

The SYBR® Premix Ex Taq (Tli RNaseH plus) qPCR kit (TaKaRa Biotechnology, Inc., Shiga, Japan) and ABI 7500 Real-Time PCR system (Applied Biosystems, Foster City, CA, USA) were used for qPCR detection. The primers are listed in Table 2. As mentioned earlier, the amplification efficiency of each primer was determined by using the double continuous dilution of cDNA (32). The amplification efficiency of GAPDH was nearly 100%. The magnification outcomes were confirmed by two methods: agarose gel electrophoresis and sequencing. As mentioned earlier, the relative expression of target gene mRNA was analyzed by the 2^−ΔΔCt^ method (33). Gene expression was normalized to GAPDH and presented as relative fold change compared to the NC group. All samples were analyzed in triplicate.

**Table 2:** Primer for real-time PCR.

**Target**	**Primer sequences (5′-3′)**	**Size, bp**	**References**
TNF-α	F: TCTATTTTGGGATCATTGCCC	127	(30)
	R: CCAGCCCCTCATTCTCTTTCT		
IL-6	F: GATGCTTCCAATCTGGGTTCA	62	(30)
	R: CACAAGACCGGTGGTGATTCT		
TLR-2	F: TCATCTCCCAAATCTGCGAAT	167	This study
	R: GGCTGATGTTCTGAATTGACCTC		
TLR-4	F: CCGTCATTAGTGCGTCAGTTCT	100	This study
	R: TTGCAGCCCACAAAAAGCA		
TLR-5	F: CTCCTTTTTAAGCCTTGCGGATA	100	(31)
	R: TAGCATTTCCAAGGCCATGTT		
MyD88	F: GTGCCGTCGGATGGTAGT	173	This study
	R: CAGTGATGAACCGCAGGAT		
Tollip	F: TACCGTGGGCCGTCTCA	57	This study
	R: CCGTAGTTCTTCGCCAACTTG		
Bcl3	F: CGACGCGGTGGACATTAAG	73	This study
	R: ACCATGCTAAGGCTGTTGTTTTC		
GAPDH	F: GAAGGTCGGAGTGAACGGAT	150	This study
	R: CATGGGTAGAATCATACTGGACA		

*F, means forward primer; R, means reverse primer*.

### Western Blotting

The intestinal mucosa samples (50–100 mg; *n* = 6) were homogenized in 1 mL of lysis buffer that was supplemented with protease and phosphatase inhibitors and centrifuged at 12,000 × g for 15 min at 4°C, and the supernatants were used for Western blotting analysis. Protein concentrations were determined using a BCA protein assay kit (Pierce Chemical Co., Rockford, IL, USA). The following primary antibodies were used: mouse anti-IκBα (Cell Signaling Technology, #MA5-15132, 1:1,000 dilution), mouse anti-phospho-IκBα MAb (Sigma-Aldrich, #MA5-15224, 1:500 dilution), rabbit polyclonal anti-NF-κB p65 (Abcam, ab90532, 1:2,000 dilution), rabbit anti-phospho-NF-κB p65 MAb (Cell Signaling Technology, #3033, 1:500 dilution), and mouse anti-GAPDH MAb (Thermo Fisher, ab10977387, 1:5,000 dilution). Horseradish peroxidase-conjugated AffiniPure goat anti-mouse IgG (H+L) (Jackson, #115-035-003, 1:10,000 dilution) or goat anti-rabbit IgG (H+L) (Jackson, #111-035-003, 1:10,000 dilution) were used as secondary antibodies. In order to reduce the variation between the gels, a sample collected from each treatment group after 3 h was repeatedly detected in the same gel. A total of 18 samples from the three treatment groups (*n* = 6, each piglet as a sample) were detected simultaneously in four gels. Gel Pro analyzer was used to analyze the optical density, and GAPDH was used to correct the protein loading. The same processing was followed for 6 and 12 h samples.

### Histological Evaluation

The jejunum segments fixed in 4% neutral formalin solution were dehydrated, embedded, and stained with hematoxylin and eosin (H&E) and examined according to previously described methods (34). All analyses of the intestinal morphology were executed by the same person.

### Statistical Analysis

All data were expressed as mean ± standard error of the mean (SEM) and were subjected to one-way ANOVA (SASInstitute Inc., Cary, NC, 2002) with each individual piglet considered as an experimental unit. The Turkey multiple comparison test was used to determine differences among the means of treatment groups. A probability value of <0.05 or <0.01 was considered statistically significant.

## Results

### Immune Status

As shown in Table 3, the serum concentrations of CRP, TNF-α, and IL-6 were assessed, as well as the mRNA expressions of TNF-α and IL-6 in piglet jejunal mucosa were examined. There was an increase in CRP and TNF-α in the PC group relative to the NC group (*P* < 0.01). Compared to the PC group, the CRP and TNF-α levels in serum at each time point decreased obviously after CB supplementation (*P* < 0.01). TNF-α mRNA expression with the same trend was detected in the PC group compared to the NC group at 3, 6, and 12 h after ETEC K88 challenge (*P* < 0.01), and a significant decrease in TNF-α mRNA expression was observed in the PC+CB group compared to the PC group (*P* < 0.01). ETEC K88 treatment could increase the serum IL-6 content and mRNA expression in the jejunum compared to the NC group, however, CB supplementation attenuated such enhancement moderately.

**Table 3:** Effect of CB on immune status in ETEC K88-infected piglets^a^.

**Item**	**NC**	**PC**	**PC + CB**	**Contrast (*****P*****-value)**^**b**^		
				**1#**	**#2**	**#3**
**CRP, ng/L**						
3 h	2.24 ± 0.11	6.92 ± 0.14	3.08 ± 0.10	<0.001	<0.001	<0.001
6 h	2.13 ± 0.10	16.10 ± 0.31	5.89 ± 0.17	<0.001	<0.001	<0.001
12 h	2.15 ± 0.12	17.11 ± 0.30	5.76 ± 0.20	<0.001	<0.001	<0.001
**TNF-α, ng/L**						
3 h	37.74 ± 2.49	95.50 ± 7.96	70.41 ± 2.56	<0.001	<0.001	0.003
6 h	37.02 ± 2.50	182.19 ± 15.79	88.74 ± 7.30	<0.001	0.003	<0.001
12 h	37.38 ± 2.20	137.59 ± 13.37	73.21 ± 2.45	<0.001	0.006	<0.001
**TNF-α, fold change**						
3 h	1.00 ± 0.06	1.48 ± 0.07	1.35 ± 0.04	<0.001	0.003	<0.001
6 h	1.00 ± 0.07	3.32 ± 0.12	1.67 ± 0.06	<0.001	<0.001	<0.001
12 h	1.00 ± 0.04	1.67 ± 0.07	1.30 ± 0.04	<0.001	0.001	<0.001
**IL-6, ng/L**						
3 h	166.50 ± 7.22	181.35 ± 9.09	174.57 ± 10.05	0.256	0.525	0.603
6 h	168.52 ± 7.78	268.46 ± 19.75	225.52 ± 16.45	<0.001	0.015	0.058
12 h	168.38 ± 5.96	227.57 ± 13.03	199.16 ± 5.65	<0.001	0.027	0.041
**IL-6, fold change**						
3 h	1.00 ± 0.08	1.10 ± 0.27	1.17 ± 0.22	0.429	0.222	0.650
6 h	1.00 ± 0.08	2.84 ± 0.50	2.02 ± 0.32	0.002	0.057	0.117
12 h	1.00 ± 0.04	1.59 ± 0.22	1.32 ± 0.15	0.018	0.169	0.241

a *NC, piglets were fed the daily diet and received oral administration of sterile physiological saline; PC, piglets were fed the daily diet and received oral challenge with ETEC; PC + CB, piglets were fed the CB-supplemented diet and received oral challenge with ETEC K88. Values are means ± standard error*.

b *Contrast, #1:NC vs. PC; #2: NC vs. PC + CB; #3: PC vs. PC + CB*.

### Effect of CB on the Expression of Toll-Like Receptors in Piglet Jejuna

As shown in Table 4, the mRNA expressions of TLR-2, TLR-4, and TLR-5 were assessed. At each time point after ETEC K88 challenge, qPCR showed an increase in TLR-2, TLR-4, and TLR-5 in the PC group relative to the NC group (*P* < 0.01). Compared to the PC group, the TLR-2, TLR-4, and TLR-5 levels decreased in the PC + CB group (TLR-2: *P* < 0.05; TLR-4: *P* < 0.01; TLR-5: *P* < 0.05).

**Table 4:** Effect of CB on the mRNA expression of TLR-2, TLR-4, and TLR-5 in ETEC K88-infected piglets^a^.

**Item**	**NC**	**PC**	**PC + CB**	**Contrast (*****P*****-value)^b^**		
				**#1**	**#2**	**#3**
**TLR-2, fold change**						
3 h	1.00 ± 0.02	2.80 ± 0.29	1.94 ± 0.15	<0.001	0.003	0.006
6 h	1.00 ± 0.06	3.69 ± 0.35	2.98 ± 0.21	<0.001	<0.001	0.014
12 h	1.00 ± 0.02	4.36 ± 0.19	3.52 ± 0.37	<0.001	<0.001	0.027
**TLR-4, fold change**						
3 h	1.00 ± 0.04	4.71 ± 0.43	2.52 ± 0.25	<0.001	0.002	<0.001
6 h	1.00 ± 0.04	7.00 ± 0.60	3.32 ± 0.27	<0.001	0.001	<0.001
12 h	1.00 ± 0.02	9.02 ± 0.66	4.25 ± 0.40	<0.001	<0.001	<0.001
**TLR-5, fold change**						
3 h	1.00 ± 0.05	1.96 ± 0.14	1.58 ± 0.09	<0.001	0.001	0.017
6 h	1.00 ± 0.05	2.83 ± 0.41	2.34 ± 0.23	<0.001	0.003	0.029
12 h	1.00 ± 0.03	3.57 ± 0.36	2.85 ± 0.30	<0.001	<0.001	0.013

a *NC, piglets were fed the daily diet and received oral administration of sterile physiological saline; PC, piglets were fed the daily diet and received oral challenge with ETEC K88; PC + CB, piglets were fed the CB-supplemented diet and received oral challenge with ETEC K88. Values are means ± standard error*.

b *Contrast, #1:NC vs. PC; #2: NC vs. PC + CB; #3: PC vs. PC + CB*.

### Effect of CB on the Activation of NF-κB p65 and IκBα in Piglet Jejuna

**Figure 1:**
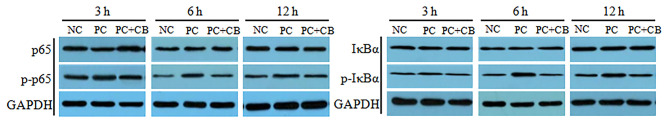
Effect of CB on the activation of NF-κB p65 and IκBα in ETEC K88-infected piglets. Pigs were challenged with or without ETEC (1 × 10^9^ CFU/kg BW). At 3, 6, and 12 h post inflammatory challenge, intestinal mucosa samples were collected to examine the expression of p65, p-p65, IκBα, and p-IκBα by Western blotting. NC, piglets were fed the daily diet and received oral administration of sterile physiological saline; PC, piglets were fed the daily diet and received oral challenge with ETEC K88; PC + CB, piglets were fed the CB-supplemented diet and received oral challenge with ETEC K88.

The activation of NF-κB p65 and IκBα in swine intestines was examined by Western blotting (Figure 1). A densitometric result was demonstrated and is shown in Table 5. Compared to the NC group, there was no significant difference in the phosphorylation of NF-κB p65 when ETEC K88 had been tested for 3 h in the PC group, but a higher phosphorylation of NF-κB p65 was observed in the PC group when ETEC K88 had been tested for 6 and 12 h, respectively (*P* < 0.01). ETEC K88-activated NF-κB p65 phosphorylation was attenuated by pretreatment with CB at 6 h post challenge (*P* < 0.05).

**Table 5:** Effect of CB on the activation of NF-κB p65 and IκBα in ETEC K88-infected piglets^a^.

**Item**	**NC**	**PC**	**PC + CB**	**Contrast (*****P*****-value)^b^**		
				**#1**	**#2**	**#3**
**p-p65, fold change**						
3 h	1.00 ± 0.06	1.22 ± 0.13	1.03 ± 0.07	0.113	0.830	0.164
6 h	1.00 ± 0.05	2.05 ± 0.28	1.45 ± 0.17	0.002	0.116	0.045
12 h	1.00 ± 0.04	1.53 ± 0.15	1.31 ± 0.14	0.007	0.095	0.208
**p-IκBα, fold change**						
3 h	1.00 ± 0.06	1.31 ± 0.08	1.04 ± 0.07	0.027	0.717	0.015
6 h	1.00 ± 0.07	2.43 ± 0.20	1.62 ± 0.13	<0.001	0.009	0.001
12 h	1.00 ± 0.06	1.39 ± 0.16	1.13 ± 0.05	0.017	0.394	0.090

a *NC, piglets were fed the daily diet and received oral administration of sterile physiological saline; PC, piglets were fed the daily diet and received oral challenge with ETEC K88; PC + CB, piglets were fed the CB-supplemented diet and received oral challenge with ETEC K88. Values are means ± standard error*.

b *Contrast, #1:NC vs. PC; #2: NC vs. PC + CB; #3: PC vs. PC + CB*.

Compared to the NC group, a higher activation of IκBα was observed in Pthe C group at each time point after ETEC K88 challenge (*P* < 0.05 or *P* < 0.01), but this provocation of ETEC K88 which experienced an increase after IκBα initiation was weakened by CB when ETEC K88 (*P* < 0.05 and *P* < 0.01) had been tested after 3 and 6 h, respectively.

### Effect of CB on the mRNA Expression of Key Proteins Involved in TLRs/NF-κB Inflammatory Signaling

The mRNA expression levels of key proteins involved in TLRs/NF-κB inflammatory signaling are shown in Table 6. Compared to the NC group, an increase in the mRNA expression of the MyD88 was observed in the PC group at each time point (*P* < 0.01). There was no difference in MyD88 expression level between the PC group and PC + CB group at 3, 6, and 12 h after ETEC K88 challenge (*P* > 0.05). Compared to the NC group, a decrease in the mRNA expression of the Tollip was observed in the PC group at each time point (*P* < 0.01). But CB pretreatment alleviated the decrease of Tollip induced by ETEC K88 at 3, 6, and 12 h (*P* < 0.01). Compared to the NC group, there was no difference in Bcl3 expression level in the PC group at 3, 6, and 12 h after challenge (*P* > 0.05). Compared to the PC group, however, an obvious increase in Bcl3 expression in the PC+CB group was observed at each time point (*P* < 0.05).

**Table 6:** Effect of CB on the mRNA expression of key proteins in TLRs/NF-κB inflammatory signaling^a^.

**Item**	**NC**	**PC**	**PC + CB**	**Contrast (*****P*****-value)^b^**		
				**#1**	**#2**	**#3**
**MyD88, fold change**						
3 h	1.00 ± 0.04	2.76 ± 0.14	2.91 ± 0.23	<0.001	<0.001	0.509
6 h	1.00 ± 0.08	4.24 ± 0.32	4.81 ± 0.25	<0.001	<0.001	0.112
12 h	1.00 ± 0.08	5.52 ± 0.29	5.57 ± 0.25	<0.001	<0.001	0.886
**Tollip, fold change**						
3 h	1.00 ± 0.06	0.28 ± 0.04	1.38 ± 0.07	<0.001	<0.001	<0.001
6 h	1.00 ± 0.07	0.42 ± 0.05	1.70 ± 0.10	<0.001	<0.001	<0.001
12 h	1.00 ± 0.07	0.52 ± 0.06	2.13 ± 0.14	0.003	<0.001	<0.001
**BCl3, fold change**						
3 h	1.00 ± 0.06	0.90 ± 0.08	1.35 ± 0.13	0.476	0.017	0.004
6 h	1.00 ± 0.07	1.14 ± 0.09	1.95 ± 0.16	0.403	<0.001	<0.001
12 h	1.00 ± 0.04	1.29 ± 0.09	2.38 ± 0.27	0.255	<0.001	0.002

a *NC, piglets were fed the daily diet and received oral administration of sterile physiological saline; PC, piglets were fed the daily diet and received oral challenge with ETEC K88; PC + CB, piglets were fed the CB-supplemented diet and received oral challenge with ETEC K88. Values are means ± standard error*.

b *Contrast, #1:NC vs. PC; #2: NC vs. PC + CB; #3: PC vs. PC + CB*.

### Changes in Intestinal Morphology

**Figure 2:**
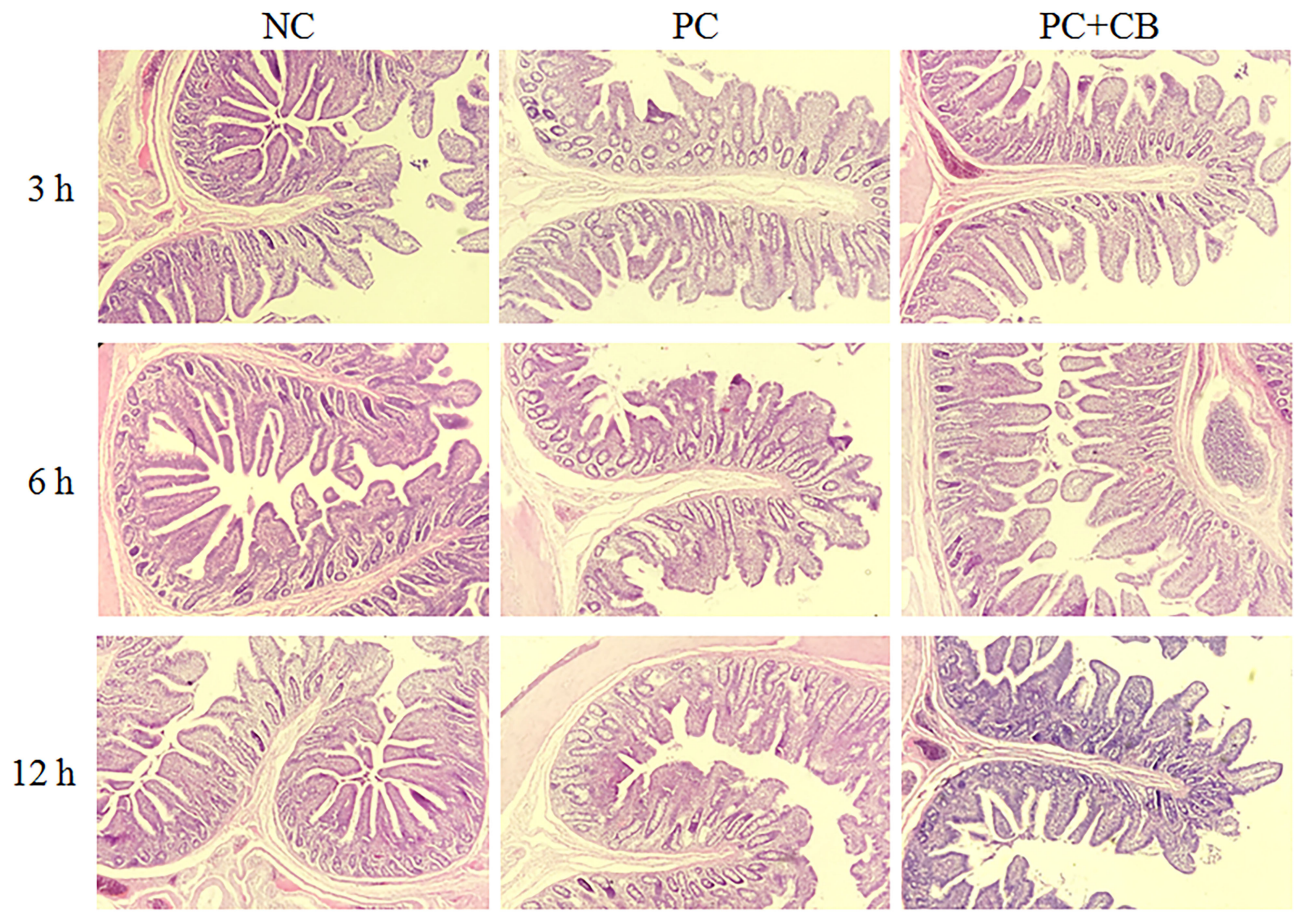
Effect of CB on the jejunal morphology of piglets. Pigs were challenged with or without ETEC (1 × 10^9^ CFU/kg BW). At 3, 6, and 12 h post inflammatory challenge, jejunal samples were collected for histopathological examination. NC, piglets were fed the daily diet and received oral administration of sterile physiological saline; PC, piglets were fed the daily diet and received oral challenge with ETEC K88; PC + CB, piglets were fed the CB-supplemented diet and received oral challenge with ETEC K88.

The morphology of piglet jejunum is shown in Figure 2 and Table 7. It was observed that ETEC K88 caused jejunal mucosal injury, including short villi and deep crypts. The *C. butyricum* supplementation alleviated the intestinal mucosal injury caused by ETEC K88. The ETEC K88 challenge at 12 h increased crypt depth (*P* < 0.01) and reduced the ratio of villus height to crypt depth (*P* < 0.01) compared to the NC group. When ETEC K88 had been tested for 12 h, the *C. butyricum* supplementation reduced the crypt depth (*P* = 0.002) compared to the PC group. The ratio of villus height to crypt depth in the PC + CB group was markedly increased than that in the PC group (*P* < 0.05 or *P* < 0.01).

**Table 7:** Effect of CB on intestinal morphology of piglet jejunum^a^.

**Item**	**NC**	**PC**	**PC + CB**	**Contrast (*****P*****-value)^b^**		
				**#1**	**#2**	**#3**
**Villus height**, **μm**						
3 h	342 ± 23	332 ± 22	353 ± 13	0.707	0.716	0.462
6 h	339 ± 22	327 ± 24	345 ± 15	0.703	0.838	0.559
12 h	340 ± 22	299 ± 13	341 ± 14	0.109	0.967	0.101
**Crypt depth**, **μm**						
3 h	133 ± 7	144 ± 7	129 ± 3	0.193	0.615	0.080
6 h	135 ± 6	151 ± 7	133 ± 10	0.161	0.821	0.109
12 h	133 ± 6	173 ± 5	144 ± 5	<0.001	0.197	0.002
**Villus height: crypt depth**						
3 h	2.54 ± 0.12	2.20 ± 0.10	2.74 ± 0.09	0.036	0.181	0.002
6 h	2.51 ± 0.12	2.06 ± 0.10	2.68 ± 0.23	0.032	0.479	0.041
12 h	2.55 ± 0.09	1.74 ± 0.10	2.38 ± 0.08	<0.001	0.207	<0.001

a *NC, piglets were fed the daily diet and received oral administration of sterile physiological saline; PC, piglets were fed the daily diet and received oral challenge with ETEC K88; PC + CB, piglets were fed the CB-supplemented diet and received oral challenge with ETEC K88. Values are means ± standard error*.

b *Contrast, #1:NC vs. PC; #2: NC vs. PC + CB; #3: PC vs. PC + CB*.

## Discussion

There is growing evidence that CB is beneficial for the treatment of intestinal inflammatory disorders. It has been verified that, in some cases, probiotics mediate the protection against pathogens and chemical-induced inflammation and inflammatory bowel disease by regulating the balance between pro-inflammatory and anti-inflammatory cytokine production in immune cells (13, 17–19, 35). Weaning is an important transitional stage in the entire life of pigs because they are suddenly forced to adapt to nutritional and environmental changes, which provoke host proinflammatory immune responses and induce gut inflammatory and metabolic diseases (18). However, reports on the use of CB in the alleviation pf intestinal inflammatory disorders and for the protection of piglets against inflammatory responses due to intestinal infection are limited. Therefore, in our research, piglets were employed as experimental inflammation models and orally challenged with ETEC K88 to investigate the effects of CB on inflammatory response, as well as to explicate the mechanisms related to anti-inflammatory activities.

As a sensitive marker of inflammatory injury, CRP is normally low in serum. The CRP concentrations of the control piglets were in accordance with previous reports (36, 37). In the inflammatory stimuli, the CRP content in serum can rapidly rise by several hundred or even a thousand times compared to normal values (38, 39). Increases in cytokines, such as TNF-α and IL-1β, are known to lead to CRP production (40). Therefore, these molecules can be used as a proper auxiliary diagnosis method of infection (38). Our results showed that CB could increase the content of CRP but had no significant effect compared to the NC group. We also observed that CB downregulated the levels of CRP in the serum of piglets challenged with ETEC K88. From these data, we could speculate that CB is able to attenuate the inflammatory reaction caused by ETEC K88.

Cytokines have a wide range of functions, such as the regulation of innate and adaptive immunity, production of blood cells, cell growth, and tissue repair. Several studies have shown that CB regulates the production of cytokines, including TNF-α, IL-6, IL-12, IL-10, and TGF-β in cells (12, 17, 18, 35, 41, 42). In the present study, we observed that CB downregulated the levels of TNF-α and IL-6 in the serum of piglets challenged with ETEC K88. These results coincided with the findings of previous reports that CB imparted anti-inflammatory effects by downregulating the production of pro-inflammatory cytokines (41, 43). Our findings indicate that CB can alleviate the ETEC K88-induced production of proinflammatory cytokines in piglets, especially after challenge with ETEC K88 for 6 and 12 h.

The ratio of villus height to crypt depth is an important morphological parameter and is considered as a useful criterion for assessing intestinal health and function (15, 32). In previous studies, it was demonstrated that oral administration of ETEC in piglets within 1–12 h can cause inflammatory response, leading to intestinal morphological damage and intestinal barrier function damage (29, 44, 45). Hence, the time points of the third, sixth, and twelfth hour were selected. Histological analysis showed that CB increased the villus/crypt ratio in control piglets, whereas this decreased obviously in piglets challenged with ETEC K88 after 12 h. Broiler chickens tested with ETEC K88 and fed on a diet supplemented with CB shared the same result (15). Several cytokines influence intestinal function. For example, IL-6 is related to the change of the intestinal tight junction caused by pathogens (46). Furthermore, TNF-α has remarkable dual functionality, as it is involved in both tissue regeneration and destruction (46). Real-time PCR showed that CB downregulated the mRNA levels of TNF-α and IL-6 in the jejunal mucosa of piglets challenged with ETEC K88. It has been previously reported that metabolites produced by probiotic bacterial strains improve the ecosystem of the intestinal tract by promoting the growth of healthy symbionts and/or enhancing the barrier function of epithelial cells (18). Therefore, our results have demonstrated that administration of the probiotic bacterial strain CB attenuates ETEC K88-induced intestinal inflammation by improving the morphology of the jejunum and reducing the expression of pro-inflammatory cytokines.

TLRs initiate the inflammatory response by sensing PAMPs, while they also play pivotal roles in the regulation of intestinal homeostasis and inflammation (18, 20, 24, 47–49). Inflammation-induced intestinal damage might be triggered by pathogens with TLRs, leading to an abdominal epithelium wound and damaged resistance to PAMPs which might be caused by an unsuitable TLR sign (25, 50). Probiotics have been found to enhance innate immunity and modulate pathogen-induced inflammation via Toll-like receptor–regulated signaling pathways (25, 51). Although recent evidence has shown that TLR-2, TLR-4, and TLR-5 favor the production of pro-inflammatory cytokines (20, 24, 52, 53), whether the TLRs participate in the inflammatory response induced by ETEC K88, and whether CB can regulate the expression of these TLRs remains unclear. In this study, it has been found that CB does lower the mRNA expression of TLR-2, TLR-4, and TLR-5 in research subjects with ETEC K88. It has been reported that oral treatment of colitis with the TLR-2 ligand proprotein convertase subtilisin kexin (PCSK) suppresses mucosal inflammation *in vivo* (54). Another report showed that CB enhances the levels of proinflammatory cytokines, such as TNF-α, IL-6, and IL-8, in HT-29 cells through TLR-2 in the absence of a pathogen (47). It has been previously reported that CB induces high levels of TLR-2 mRNA and protein expression and moderate levels of TNF-α production in silvery pomfret intestinal epithelial cells (16). Furthermore, the production of IL-10, an anti-inflammatory cytokine that is secreted in the presence of CB in intestinal macrophages of the inflammatory mucosa of colitis mice, is almost completely abolished in the absence of TLR-2 (18). Therefore, our results indicate that TLR-2 and TLR-4 may be involved in the molecular interactions between the host and bacteria that contribute to a reduction in intestinal inflammation in piglets infected with ETEC K88 by decreasing proinflammatory cytokine production.

TLRs activate NF-κB by affecting a variety of lower-reach intracellular genes (20, 22, 48, 52). It is well-known that IκBα protein phosphorylation and its subsequent degradation are responsible for p65 nuclear translocation (8). NF-κB pathway activation regulated by IKKα and IκBα plays an essential role in regulating inflammatory cytokines release, such as TNF-α, IL-1β, and IL-6 (22, 55). Activated NF-κB p65 stimulates the synthesis of anti- and pro-inflammatory cytokines, including IL-6, IL-1β, TNF-α, and IL-10 (26, 56). In the present study, we further explored whether NF-κB p65 and IκBα affect the control of CB-mediated intestinal inflammation of piglets infected with ETEC K88. Our results demonstrated that ETEC K88 increased the phosphorylation levels of both NF-κB p65 and IκBα–counteracted by CB. A similar result was reported *in vitro*, where pretreatment of HT-29 cells with CB reduced the expression TNF-α and NF-κB p65 following *S. aureus* lipoteichoic acid (aLTA)-induced inflammation, compared to control cell lines (17). Our results showed that ETEC K88 induced the expression of CRP, TNF-α, and IL-6 in piglets, as well as activated NF-κB p65 and IκBα; therefore, downregulation of the upstream signaling protein, IκBα, may be involved in CB-mediated inhibition of ETEC K88-induced NF-κB activation. Our data indicate that the suppression of phosphorylation of NF-κB p65 and IκBα by CB might inhibit pro-inflammatory cytokines expression.

Negative regulatory mechanisms play an important role in weakening and maintaining the TLR signaling pathway (57). *L. jensenii* TL2937 has been reported to lower LPS-causing proinflammatory cytokines and chemokine production in PIE cells by improving the expression of Bcl-3 and mitogen-activated protein kinase phosphatase-1 (MKP-1) which slow down TLR-4-dependent NF-κB activation (25). Another example is that the continuous upregulation of Tollip and SIGIRR have an important impact on the anti-inflammatory activity of *L. casei* MEP221114 in poly (I:C)-stimulated PIE cells (58). However, whether CB regulates the inhibitory factors of TLR signaling in an ETEC K88-induced inflammatory reaction remains unclear. Our results show that CB increases the levels of MyD88 and Bcl-3 in ETEC K88-infected piglets. It has been reported that CB-induced production of IL-10 was almost completely abolished in the absence of MyD88 (59). The findings of the present study indicate that the uptake of CB via feed results in increased production levels of negative regulators of the TLR signaling pathway, so as to build an anti-inflammatory environment to reduce inflammatory injury.

## Conclusion

In summary, it has been suggested that CB pretreatment alleviates ETEC K88-induced inflammation and intestinal damage, partly by limiting detrimental inflammatory responses via inhibition of TLR-2 and TLR-4 signaling pathways in piglets. On a theoretical basis for the clinical application of CB, this potential mechanism reduced inflammatory response.

## Data Availability Statement

The original contributions presented in the study are included in the article/supplementary material, further inquiries can be directed to the corresponding author/s.

## Author Contributions

HL conceived and designed the experiments, coordinated data curation, and wrote the original draft. XL and ZS performed the experiments. JQ reviewed and edited the manuscript. All authors contributed to the article and approved the submitted version.

## Conflict of Interest

The authors declare that the research was conducted in the absence of any commercial or financial relationships that could be construed as a potential conflict of interest.

## References

[1] Liu Y. Fatty acids, inflammation and intestinal health in pigs. J Anim Sci Biotechnol. (2015) 6:41. 10.1186/s40104-015-0040-1 26361542 PMC4564983

[2] Shirkey TW Siggers RH Goldade BG Marshall JK Drew MD Laarveld B. Effects of commensal bacteria on intestinal morphology and expression of proinflammatory cytokines in the gnotobiotic pig. Exp Biol Med. (2006) 231:1333–45. 10.1177/153537020623100807 16946402

[3] Boyer PE D'Costa S Edwards LL Milloway M Susick E Borst LB. Early-life dietary spray-dried plasma influences immunological and intestinal injury responses to later-life *Salmonella* typhimurium challenge. Br J Nutr. (2015) 113:783–93. 10.1017/S000711451400422X 25671331 PMC4382447

[4] Xiao D Wang Y Liu G He J Qiu W Hu X. Effects of chitosan on intestinal inflammation in weaned pigs challenged by enterotoxigenic *Escherichia coli*. PLoS ONE. (2014) 9:e104192. 10.1371/journal.pone.0104192 25090447 PMC4121323

[5] Scharek-Tedin L Pieper R Vahjen W Tedin K Neumann K Zentek J. *Bacillus cereus* var. Toyoi modulates the immune reaction and reduces the occurrence of diarrhea in piglets challenged with Salmonella Typhimurium DT104. J Anim Sci. (2013) 91:5696–704. 10.2527/jas.2013-6382 24126275

[6] Pié S Lallès JP Blazy F Laffitte J Sève B Oswald IP. Weaning is associated with an upregulation of expression of inflammatory cytokines in the intestine of piglets. J Nutr. (2004) 134:641–7. 10.1093/jn/134.3.641 14988461

[7] Yang Y Galle S Le MH Zijlstra RT Gänzle MG. Feed fermentation with reuteran- and levan-producing *Lactobacillus reuteri* reduces colonization of weanling pigs by enterotoxigenic *Escherichia coli*. Appl Environ Microbiol. (2015) 81:5743–52. 10.1128/AEM.01525-15 26070673 PMC4551235

[8] Fan X Zhu JY Sun Y Luo L Yan J Yang X. Evodiamine inhibits zymosan-induced inflammation *in vitro* and *in vivo*: inactivation of NF-κB by inhibiting IκBα phosphorylation. Inflammation. (2017) 40:1012–27. 10.1007/s10753-017-0546-0 28337636

[9] Pan X Wu T Song Z Tang H Zhao Z. Immune responses and enhanced disease resistance in Chinese drum, *Miichthys miiuy* (Basilewsky), after oral administration of live or dead cells of *Clostridium butyrium* CB2. J Fish Dis. (2008) 31:679–86. 10.1111/j.1365-2761.2008.00955.x 18786030

[10] Yang X Zhang B Guo Y Jiao P Long F. Effects of dietary lipids and *Clostridium butyricum* on fat deposition and meat quality of broiler chickens. Poult Sci. (2010) 89:254–60. 10.3382/ps.2009-00234 20075277

[11] Zhang L Cao GT Zeng XF Zhou L Ferket PR Xiao YP. Effects of *Clostridium butyricum* on growth performance, immune function, and cecal microflora in broiler chickens challenged with *Escherichia coli* K88. Poult Sci. (2014) 93:46–53. 10.3382/ps.2013-03412 24570422

[12] Wang FY Liu JM Luo HH Liu AH Jiang Y. Potential protective effects of *Clostridium butyricum* on experimental gastric ulcers in mice. World J Gastroenterol. (2015) 21:8340–51. 10.3748/wjg.v21.i27.8340 26217085 PMC4507103

[13] Kashiwagi I Morita R Schichita T Komai K Saeki K Matsumoto M. Smad2 and Smad3 inversely regulate TGF- autoinduction in *Clostridium butyricum*-activated dendritic cells. Immunity. (2015) 43:65–79. 10.1016/j.immuni.2015.06.010 26141582

[14] Liao XD Ma G Cai J Fu Y Yan XY Wei XB. Effects of *Clostridium butyricum* on growth performance, antioxidation, and immune function of broilers. Poult Sci. (2015) 94:662–7. 10.3382/ps/pev038 25717087

[15] Zhang L Zhang L Zhan X Zeng X Zhou L Cao G. Effects of dietary supplementation of probiotic, *Clostridium butyricum*, on growth performance, immune response, intestinal barrier function, and digestive enzyme activity in broiler chickens challenged with *Escherichia coli* K88. J Anim Sci Biotechnol. (2016) 7:3. 10.1186/s40104-016-0061-4 26819705 PMC4728939

[16] Gao Q Xiao Y Zhang C Min M Peng S Shi Z. Molecular characterization and expression analysis of toll-like receptor 2 in response to bacteria in silvery pomfret intestinal epithelial cells. Fish Shellfish Immunol. (2016) 58:1–9. 10.1016/j.fsi.2016.08.057 27574826

[17] Wang JB Qi LL Mei LH Wu ZG Wang HZ. *C. butyricum* lipoteichoic acid inhibits the inflammatory response and apoptosis in HT-29 cells induced by *S. aureus* lipoteichoic acid. Int J Biol Macromol. (2016) 88:81–7. 10.1016/j.ijbiomac.2016.03.054 27020942

[18] Hayashi A Sato T Kamada N Mikami Y Matsuoka K Hisamatsu T. A single strain of *Clostridium butyricum* induces intestinal IL-10-producing macrophages to suppress acute experimental colitis in mice. Cell Host Microbe. (2013) 13:711–22. 10.1016/j.chom.2013.05.013 23768495

[19] Cai M Zeng L Li LJ Mo LH Xie RD Feng BS. Specific immunotherapy ameliorates ulcerative colitis. Allergy Asthma Clin Immunol. (2016) 12:37. 10.1186/s13223-016-0142-0 27499766 PMC4975874

[20] Qin Y Li H Qiao J. TLR2/MyD88/NF-κB signaling pathway regulates IL-8 production in porcine alveolar macrophages infected with porcine circovirus 2. J Gen Virol. (2016) 97:445–52. 10.1099/jgv.0.000345 26581603

[21] Kasinski AL Du Y Thomas SL Zhao J Sun SY Khuri FR. Inhibition of IκB kinase-nuclear factor-κB signaling pathway by 3,5-bis (2-flurobenzylidene) piperidin-4-one (EF24), a novel monoketone analog of curcumin. Mol Pharmacol. (2008) 74:654–61. 10.1124/mol.108.046201 18577686 PMC2638506

[22] Han X Wu YC Meng M Sun QS Gao SM Sun H. Linarin prevents LPS-induced acute lung injury by suppressing oxidative stress and inflammation via inhibition of TXNIP/NLRP3 and NF-κB pathways. Int J Mol Med. (2018) 42:1460–72. 10.3892/ijmm.2018.3710 29845284 PMC6089707

[23] Jiang Y Lü X Man C Han L Shan Y Qu X. *Lactobacillus acidophilus* induces cytokine and chemokine production via NF-κB and p38 mitogen-activated protein kinase signaling pathways in intestinal epithelial cells. Clin Vaccine Immunol. (2012) 19:603–8. 10.1128/CVI.05617-11 22357649 PMC3318281

[24] Li H Zhang L Chen L Zhu Q Wang W Qiao J. *Lactobacillus acidophilus* alleviates the inflammatory response to enterotoxigenic *Escherichia coli* K88 via inhibition of the NF-κB and p38 mitogen-activated protein kinase signaling pathways in piglets. BMC Microbiol. (2016) 16:273–80. 10.1186/s12866-016-0862-9 27832756 PMC5105324

[25] Shimazu T Villena J Tohno M Fujie H Hosoya S Shimosato T. Immunobiotic *Lactobacillus jensenii* elicits anti-inflammatory activity in porcine intestinal epithelial cells by modulating negative regulators of the Toll-like receptor signaling pathway. Infect Immun. (2012) 80:276–88. 10.1128/IAI.05729-11 22083706 PMC3255675

[26] Liu J Fu Y Zhang H Wang J Zhu J Wang Y. The hepatoprotective effect of the probiotic *Clostridium butyricum* against carbon tetrachloride-induced acute liver damage in mice. Food Funct. (2017) 8:4042–52. 10.1039/C7FO00355B 28933492

[27] Cerf-Bensussan N Gaboriau-Routhiau V. The immune system and the gut microbiota: friends or foes? Nat Rev Immunol. (2010) 10:735–44. 10.1038/nri2850 20865020

[28] Shibolet O Podolsky DK TLRs in the Gut. IV. Negative regulation of Toll-like receptors and intestinal homeostasis: addition by subtraction. Am J Physiol Gastrointest Liver Physiol. (2007) 292:1469–73. 10.1152/ajpgi.00531.2006 17554134

[29] Li HH Li YP Zhu Q Qiao JY Wang WJ. Dietary supplementation with *Clostridium butyricum* helps to improve the intestinal barrier function of weaned piglets challenged with enterotoxigenic *Escherichia coli* K88. J Appl Microbiol. (2018) 125:964–75. 10.1111/jam.13936 29851202

[30] Chen L Li S Zheng J Li W Jiang X Zhao X. Effects of dietary *Clostridium butyricum* supplementation on growth performance, intestinal development, and immune response of weaned piglets challenged with lipopolysaccharide. J Anim Sci Biotechnol. (2018) 9:62. 10.1186/s40104-018-0275-8 30159141 PMC6106813

[31] Bi J Song S Fang L Wang D Jing H Gao L. Porcine reproductive and respiratory syndrome virus induces IL-1β production depending on TLR4/MyD88 pathway and NLRP3 inflammasome in primary porcine alveolar macrophages. Mediators Inflamm. (2014) 2014:403515. 10.1155/2014/403515 24966466 PMC4055429

[32] Liu Y Chen F Odle J Lin X Jacobi SK Zhu H. Fish oil enhances intestinal integrity and inhibits TLR4 and NOD2 signaling pathways in weaned pigs after LPS challenge. J Nutr. (2012) 142:2017–24. 10.3945/jn.112.164947 23014495

[33] Zhu YH Li XQ Zhang W Zhou D Liu HY Wang JF. Dose-dependent effects of *Lactobacillus rhamnosus* on serum interleukin-17 production and intestinal T-Cell responses in pigs challenged with *Escherichia coli*. Appl Environ Microbiol. (2014) 80:1787–98. 10.1128/AEM.03668-13 24389928 PMC3957626

[34] Li H Wang Y Ding L Zheng SJ. *Staphylococcus sciuri* exfoliative toxin C (ExhC) is a necrosis-inducer for mammalian cells. PLoS ONE. (2011) 6:e23145. 10.1371/journal.pone.0023145 21829591 PMC3146541

[35] Xu LZ Yang LT Qiu SQ Yang G Luo XQ Miao BP. Combination of specific allergen and probiotics induces specific regulatory B cells and enhances specific immunotherapy effect on allergic rhinitis. Oncotarget. (2016) 7:54360–9. 10.18632/oncotarget.10946 27486985 PMC5342347

[36] Heegaard PM Klausen J Nielsen JP González-Ramón N PineiroM Lampreave F. The porcine acute phase response to infection with Actinobacillus pleuropneumoniae. Haptoglobin, C-reactive protein, major acute phase protein and serum amyloid A protein are sensitive indicators of infection. Comp Biochem Physiol B Biochem Mol Biol. (1998) 119:365–73. 10.1016/S0305-0491(97)00362-3 9629669

[37] Moya SL Boyle L Lynch PB Arkins S. Pro-inflammatory cytokineand acute phase protein responses to low-dose lipopolysaccharide (LPS) challenge in pigs. Anim Sci. (2006) 82:527–34. 10.1079/ASC200665

[38] Xu X Mao W Chai Y Dai J Chen Q Wang L. Angiogenesis inhibitor, endostar, prevents vasa vasorum neovascularization in a swine atherosclerosis model. J Atheroscler Thromb. (2015) 22:1100–12. 10.5551/jat.26906 26016418

[39] Venugopal SK Devaraj S Jialal I. C-reactive protein decreases prostacyclin release from human aortic endothelial cells. Circulation. (2003) 108:1676–8. 10.1161/01.CIR.0000094736.10595.A1 14504187

[40] Ansar W Ghosh S. C-reactive protein and the biology of disease. ImmunolRes. (2013) 56:131–42. 10.1007/s12026-013-8384-0 23371836

[41] Chen CC Kong MS Lai MW Chao HC Chang KW Chen SY. Probiotics have clinical, microbiologic, and immunologic efficacy in acute infectious diarrhea. Pediatr Infect Dis J. (2010) 29:135–8. 10.1097/INF.0b013e3181b530bf 20135748

[42] Gao Q Qi L Wu T Wang J. An important role of interleukin-10 in counteracting excessive immune response in HT-29 cells exposed to *Clostridium butyricum*. BMC Microbiol. (2012) 12:100. 10.1186/1471-2180-12-100 22681958 PMC3410821

[43] Kanauchi O Matsumoto Y Matsumura M Fukuoka M Bamba T. The beneficial effects of microflora, especially obligate anaerobes, and their products on the colonic environment in inflammatory bowel disease. Curr PharmDes. (2005) 11:1047–53. 10.2174/1381612053381675 15777254

[44] Chen HS Velayudhan DE Li A Feng Z Liu D Yin YL. Growth performance, gastrointestinal microbial activity and immunological response of piglets receiving microencapsulated *Enterococcus faecalis* CG1.0007 and enzyme complex after an oral challenge with *Escherichia coli* (K88). Can J Anim Sci. (2016) 96:609–18. 10.1139/cjas-2015-0051

[45] Thorgersen EB Hellerud BC Nielsen EW Barratt-Due A Fure H Lindstad JK. CD14 inhibition efficiently attenuates early inflammatory and hemostatic responses in *Escherichia coli* sepsis in pigs. FASEB J. (2010) 24:712–22. 10.1096/fj.09-140798 19841036 PMC2830134

[46] Zhai Z Ni X Jin C Ren W Li J Deng J. Cecropin a modulates tight junction-related protein expression and enhances the barrier function of porcine intestinal epithelial cells by suppressing the MEK/ERK pathway. Int J Mol Sci. (2018) 19:1941. 10.3390/ijms19071941 30004434 PMC6073479

[47] Gao Q Qi L Wu T Wang J. *Clostridium butyricum* activates TLR2-mediated MyD88-independent signaling pathway in HT-29 cells. Mol Cell Biochem. (2012) 361:31–7. 10.1007/s11010-011-1084-y 21956671

[48] Tomosada Y Villena J Murata K Chiba E Shimazu T Aso H. Immunoregulatory effect of bifidobacteria strains in porcine intestinal epithelial cells through modulation of ubiquitin-editing enzyme A20 expression. PLoS ONE. (2013) 8:e59259. 10.1371/journal.pone.0059259 23555642 PMC3608626

[49] Wells JM Rossi O Meijerink M Baarlen VP. Epithelial crosstalk at the microbiota–mucosal interface. Proc Natl Acad Sci USA. (2011) 108:4607–14. 10.1073/pnas.1000092107 20826446 PMC3063605

[50] Abreu MT Fukata M Arditi M. TLR signaling in the gut in health and disease. J Immunol. (2005) 174:4453–60. 10.4049/jimmunol.174.8.4453 15814663

[51] Murofushi Y Villena J Morie K Kanmani P Tohno M Shimazu T. The toll-like receptor family protein RP105/MD1 complex is involved in the immunoregulatory effect of exopolysaccharides from *Lactobacillus plantarum* N14. Mol Immunol. (2015) 64:63–75. 10.1016/j.molimm.2014.10.027 25466614

[52] Meshkibaf S Fritz J Gottschalk M Kim SO. Preferential production of G-CSF by a protein-like *Lactobacillus rhamnosus* GR-1 secretory factor through activating TLR2-dependent signaling events without activation of JNKs. BMC Microbiol. (2015) 15:238. 10.1186/s12866-015-0578-2 26502905 PMC4623291

[53] Ryu JC Kim MJ Kwon Y Oh JH Yoon SS Shin SJ. Neutrophil pyroptosis mediates pathology of *P. aeruginosa* lung infection in the absence of the NADPH oxidase NOX2. Mucosal Immunol. (2017) 10:757–74. 10.1038/mi.2016.73 27554297

[54] Cario E Gerken G Podolsky DK. Toll-like receptor 2 controls mucosal inflammation by regulating epithelial barrier function. Gastroenterology. (2007) 132:1359–74. 10.1053/j.gastro.2007.02.056 17408640

[55] Meng M. Digitoflavone (DG) attenuates LPS-induced acute lung injury through reducing oxidative stress inflammatory response dependent on the suppression of TXNIP/NLRP3 NF-κB. Biomed Pharmacother. (2017) 94:712–25. 10.1016/j.biopha.2017.07.001 28800542

[56] Lv YJ Zhang XJ Sun Y Zhang SX. Activation of NF-κB contributes to production of pig-major acute protein and serum amyloid A in pigs experimentally infected with porcine circovirus type 2. Res Vet Sci. (2013) 95:1235–40. 10.1016/j.rvsc.2013.08.006 24011594

[57] Liew FY Xu DM Brint EK O'Neill LAJ. Negative regulation of toll-like receptor-mediated immune responses. Nat Rev Immunol. (2005) 5:446–58. 10.1038/nri1630 15928677

[58] Hosoya S Villena J Chiba E Shimazu T Suda Y Aso H. Advanced application of porcine intestinal epithelial cells for the selection of immunobiotics modulating toll-like receptor 3-mediated inflammation. J Microbiol Immunol Infect. (2013) 46:474–81. 10.1016/j.jmii.2012.04.005 22727542

[59] Qiao J Li H Wang Z Wang W. Effects of *Lactobacillus acidophilus* dietary supplementation on the performance, intestinal barrier function, rectal microflora and serum immune function in weaned piglets challenged with *Escherichia coli* lipopolysaccharide. Antonie van Leeuwenhoek. (2015) 107:883–91. 10.1007/s10482-015-0380-z 25577203

